# Serum p-tau181 and p-tau217 levels in adolescents and adults with 5q spinal muscular atrophy

**DOI:** 10.1007/s00415-026-14194-1

**Published:** 2026-10-21

**Authors:** Caroline Deborah Stapf, Zeljko Uzelac, Louise Iterbeke, Arthur Guthknecht, Lorenzo Barba, David Strube, Anna Kölsch, Kathleen Kleeberg, Thomas Kendzierski, Andreas Roos, Petra Steinacker, Samir Abu-Rumeileh, Kristl G. Claeys, Jochen Weishaupt, Alexander Mensch, Markus Otto

**Affiliations:** 1 Department of Neurology, University Medicine Halle, Halle (Saale), Germany; 2 Department of Neurology, University of Ulm, Ulm, Germany; 3 Laboratory for Muscle Diseases and Neuropathies, Department of Neurosciences, KU Leuven, Leuven, Belgium; 4 Department of Neuropediatrics, Centre for Neuromuscular Disorders in Children, University Hospital Essen, Duisburg-Essen University, Essen, Germany; 5 Department of Neurology, University Hospitals Leuven, Leuven, Belgium; 6 Heimer Institute for Muscle Research, BG University Hospital Bergmannsheil Bochum, Bochum, Germany

**Keywords:** Spinal muscular atrophy, Phosphorylated tau (p-tau181; p-tau217), Serum biomarkers, Motor neuron disease

## Abstract

**Background:**

Reliable blood biomarkers for longitudinal monitoring in adolescent and adult 5q spinal muscular atrophy (SMA) are lacking. Phosphorylated tau (p-tau) species are elevated in serum in amyotrophic lateral sclerosis (ALS), where they may reflect peripheral nerve and muscle pathology. We aimed to investigate serum p-tau181 and p-tau217 as longitudinal biomarkers in adolescent and adult SMA and assessed associations with clinical parameters.

**Methods:**

In this retrospective multi-centre study, we assessed serum p-tau181 and p-tau217 levels in adults with 5q SMA under treatment with nusinersen or risdiplam, comparing levels with Alzheimer’s disease (AD), amyotrophic lateral sclerosis (ALS) and non-neurodegenerative disease controls.

Associations with motor function, respiratory capacity, creatine kinase (CK), and neurofilament light chain (NfL) were assessed using linear mixed-effects models adjusted for age and sex.

**Results:**

Serum p-tau181 was higher in SMA than in disease controls (*β *= −0.48, 95%-CI −0.81 to −0.15), but lower than in ALS (*β *= 0.99, 95%-CI 0.58 to 1.39) and AD (*β *= 0.56, 95%-CI 0.11 to 1.00). P-tau181 declined from baseline to FUP2 (Δ median −1.80 pg/mL, IQR −5.90 to 2.10); p-tau217 showed no longitudinal change. P-tau181 and p-tau217 were positively associated with motor function, functional status, respiratory capacity, and CK, with no association with NfL. In exploratory analyses, higher baseline p-tau181 was associated with greater HFMSE decline (*β *= 1.79, 95%-CI 0.59 to 2.99).

**Conclusions:**

Serum p-tau181 and p-tau217 appear to reflect ongoing muscle turnover in adult SMA, declining as muscle mass decreases. Higher baseline p-tau181 was associated with subsequent functional deterioration, suggesting potential utility as a biomarker of active denervation and disease monitoring.

**Supplementary Information:**

The online version contains supplementary material available at https://doi.org/10.1007/s00415-026-14194-1.

## Introduction

Autosomal recessive 5q spinal muscular atrophy (SMA) is a neurodegenerative disorder caused by loss-of-function mutations or deletions of the *SMN1* gene on chromosome 5q, leading to reduced functional SMN protein and progressive degeneration of lower motor neurons with resultant muscle weakness and atrophy [1, 2]. Clinically, SMA is classified into different subtypes. Adolescent and adult SMA primarily comprises type III and IV, which mainly correspond to patients who are, especially with type IV, highly functioning walkers and retain motoric function for several decades [1].

Three disease-modifying therapies are currently available, all aiming to increase the amount of functional SMN protein [3]. In infantile-onset SMA, the gene replacement therapy onasemnogene abeparvovec substitutes the defective *SMN1* gene and leads to substantial improvements [4]. In paediatric and adult-onset SMA, nusinersen, an intrathecally administered antisense oligonucleotide, and risdiplam, an orally available small molecule, enhance functional SMN protein production via *SMN2* pre-mRNA splicing modulation [5, 6]. Despite these advances, robust biomarkers for diagnosis and therapeutic monitoring remain lacking [7]. A gap particularly relevant in slowly progressive types III and IV, where disease duration can span decades and treatment response may be difficult to assess. Blood-based biomarkers reflecting peripheral neurodegeneration and muscle pathology could, therefore, substantially improve disease monitoring.

In recent years, neurodegenerative biomarkers have been increasingly studied in motor neuron diseases, particularly amyotrophic lateral sclerosis (ALS) [8]. Unlike SMA, ALS involves degeneration of both upper and lower motor neurons, manifests typically in the sixth to eighth decade, and follows a rapid functional decline [9]. One biomarker of interest is phosphorylated tau protein. Among its species, p-tau181 and p-tau217 are established CNS-specific early biomarkers of Alzheimer's disease (AD)-related neuropathology, measurable in both CSF and blood with comparable accuracy using highly sensitive immunoassays [10–15]. Investigations in ALS have revealed elevated blood levels of both species, with normal CSF levels [16–18]. Accordingly, increased blood p-tau may reflect protein release from injured peripheral nerves and/or denervated muscle fibres, supported by the absence of serum-CSF correlation [16, 17]. Moreover, elevated blood p-tau181 levels are associated with lower motor neuron loss in the spinal cord and clinical and electrophysiological indicators of lower motor neuron involvement [17, 18]. Recent studies additionally demonstrate the presence of both p-tau species in atrophic muscle fibres, highlighting their potential as biomarkers of muscle degeneration in ALS [16].

In SMA, p-tau variants have rarely been studied and almost exclusively in CSF with only a limited number of studies focusing on blood concentrations. To our knowledge only one study explored p-tau181 in blood (*n *= 12), which was found to be elevated in plasma samples of SMA compared to controls lacking longitudinal data [18]. A recent pre-print reported no relevant difference in serum p-tau217 between healthy controls, adult SMA, and ALS, though the analysis was restricted to a single time point, approximately 20 patients per group, and no clinical correlations [19]

We, therefore, conducted a retrospective, longitudinal, multi-centre study investigating serum p-tau181 and p-tau217 in adult SMA, evaluating longitudinal dynamics and associations with clinical parameters.

## Methods

### Inclusion and ethics

Eligible participants included patients with genetically confirmed 5q spinal muscular atrophy (SMA) (adults ≥18 years and minors <18 years), who provided written informed consent or their legal guardians. The study was conducted in accordance with Good Clinical Practice (GCP) guidelines. Ethical approval for the analysis of biomaterial and clinical data was obtained from the Ethics Committees of Martin Luther University Halle-Wittenberg, Germany (reference no. 2021-101); Ulm University, Germany (reference no. 20/10); and UZ/KU Leuven, Belgium (reference no. S69981).

### Case selection

This retrospective multi-centre study included patients with SMA (*n *= 70; Halle *n *= 9, Ulm *n *= 29, Leuven *n *= 34), ALS (*n *= 31), AD (*n *= 31), and disease controls (*n *= 31) recruited from three neurological centres (total *n* = 163). An additional exploratory cohort of eight SMA patients from Halle (*n *= 8, FUP3 only, no baseline data available) was analysed separately and is described in the Supplementary Material (Table S13–14). The diagnosis of 5q-associated SMA was genetically confirmed.

ALS, AD, and disease control patients were recruited exclusively at Martin Luther University Halle-Wittenberg and have been described previously in a recently published study [16].

ALS was diagnosed according to the revised El Escorial and Gold Coast criteria [20]. ATN status (A: Aß-proteinopathy, T: phosphorylated and secreted AD tau, N: injury, dysfunction, or degeneration of neuropil) was assessed in all ALS patients [11]; those with ATN positivity or unknown status were excluded to eliminate confounding p-tau elevation from AD co-pathology [21]. AD diagnosis required a typical CSF core biomarker profile per National Institute on Aging–Alzheimer's Association criteria [11, 22], defined in our laboratory as Aß42/40 <0.069, p-tau181 >56.5 pg/mL, and total tau >400 pg/mL. Disease controls had no evidence of neurodegeneration based on clinical evaluation, CSF analysis, and neuroimaging.

SMA data were collected longitudinally, with baseline samples obtained prior to treatment initiation. Follow-up time points were defined as follow-up 1 (FUP1, months 9–16), follow-up 2 (FUP2, months 22–26), and exploratory follow-up 3 (FUP3, months 50–58). As not all patients contributed data at every time point, analyses used all available data per time point.

Clinical variables for SMA included age, sex, disease duration, SMA type, SMN2 copy number, treatment and duration, body mass index (BMI), and motor function. Motor function was assessed using the Hammersmith Functional Motor Scale–Expanded (HFMSE), the Revised Upper Limb Module (RULM), forced vital capacity (FVC, % predicted) and the Motor Function Measure 32 (MFM32). The MFM32 was collected exclusively at the Leuven centre and was available only for patients treated with risdiplam (reflecting Belgian reimbursement requirements). In the Leuven cohort HFMSE was collected only for nusinersen-treated patients, whereas in the other cohorts HFMSE was applied for both therapies.

HFMSE, RULM, and MFM32 are well-established motor outcome measures in SMA, with higher scores indicating better motor function [23]. The HFMSE assesses gross motor function (33 items), the RULM evaluates upper limb function (20 items), and the MFM32 assesses gross and fine motor function across three domains: D1 (standing and transfer), D2 (axial and proximal), and D3 (distal motor function; 32 items total). All scores were converted to percentages of the maximum score to allow consistent comparison; absolute values are provided in the Supplementary Material.

All SMA patients received nusinersen or risdiplam at follow-up. Nusinersen (*n *= 45) was administered intrathecally at 12 mg per dose (four loading doses on days 0, 14, 28, and 63, followed by maintenance doses every four months). Risdiplam (*n *= 25) was given orally at 5 mg daily.

### Biomarker analysis

Serum samples were collected, processed, and stored at −80 °C until analysis. Sampling intervals varied by cohort and treatment: in the Halle cohort, samples were obtained at each nusinersen application and every three months during risdiplam treatment, with samples corresponding to pre-defined time points selected for analysis. In the Ulm cohort, samples were collected at baseline and after 26 months of nusinersen treatment. In the Leuven cohort, samples were collected at baseline and after 14 and 22 months of nusinersen, and at baseline and after 12 and 24 months of risdiplam treatment.

All biomarkers were measured at the Department of Neurology in University Medicine Halle. Serum p-tau181 and p-tau217 levels were quantified by single molecule array (Simoa) immunoassays on a HD-X Analyzer (Quanterix, Lexington, MA, USA) using commercially available kits. Neurofilament light chain (NfL) was measured using commercial kits on the Ella microfluidic automated ELISA system (Bio-Techne, Minneapolis, MN, USA).

Intra- and inter-assay coefficients of variation were <10% and <15%, respectively, as specified by the manufacturer. Further details are provided in the Supplementary Information.

### Statistical analysis

Statistical analyses were performed using GraphPad Prism 10 (GraphPad Software Inc., Boston, Massachusetts, USA) and R 4.5.2 (packages *lme4* and *lmerTest,* R Foundation for Statistical Computing, Vienna, Austria).

Data are presented as mean ± SD or median (IQR). Non-parametric group comparisons were performed using Mann–Whitney U or Kruskal–Wallis tests with Dunn’s post-hoc correction. Paired longitudinal comparisons used the Wilcoxon signed-rank test.

Group differences across diagnostic categories were assessed using multiple linear regression with diagnostic group as a categorical predictor and age and sex as covariates, with SMA as the reference category. Associations between biomarker levels and clinical variables were evaluated using linear mixed-effects models (LME) with patient ID as a random intercept, adjusted for age and sex. Study center was included as an additional fixed effect where applicable. Biomarker concentrations were naturally log-transformed to improve normality of residuals. For categorical predictors with more than two levels, global effects were assessed using type III Wald or likelihood ratio tests. To account for multiple testing, *p* values were adjusted using the Benjamini–Hochberg false discovery rate procedure.

Spearman's rank correlation was used to assess associations between variables; where indicated, linear regression was performed following log-transformation of the dependent variable. To assess whether baseline p-tau levels predicted motor function change, multivariable linear regression models were calculated with ΔRULM and ΔHFMSE (baseline to FUP2) as dependent variables and baseline p-tau181 or p-tau217 (ln-transformed), baseline motor score, age, and sex as independent variables, with separate models per biomarker and outcome.

As BMI was unavailable for all patients and showed no relevant association with serum p-tau in exploratory analyses (p-tau181: *ρ *= 0.12; *p*-tau217: *ρ *= 0.14; Table S15), models were adjusted for age and sex only.

Due to incomplete longitudinal data, comparisons across time points used available-case analyses. Analyses involving MFM32 were considered exploratory owing to limited sample size and single-centre restriction.

GraphPad Prism was used for visualisation of associations identified in LME models and for additional exploratory analyses not intended to be confirmatory. Statistical significance was defined as a two-sided *p* value <0.05.

## Results

### Demographic and clinical characteristics of the study group

Demographic and clinical characteristics are summarised in Table 1.

**Table 1:** Demographic characteristics and serum biomarker levels across diagnostic groups

	SMA (Baseline)	Disease controls	ALS	AD	*p*-value
*n*	70	31	31	31	
Age [years], median (IQR)	35.00 (24.00–46.50)	46.00 (36.00–53.00)	66.00 (57.00–71.00)	71.00 (65.00–73.00)	**<0.001**
Female, *n* (%)	29 (37.17)	14 (45.16)	6 (19.35)	13 (41.94)	0.084
p-tau181 [pg/mL], median (IQR)	19.69 (7.30–26.20)	8.98 (7.01–10.81)	47.53 (26.52–61.30)	27.16 (20.31–31.81)	**<0.001** (model p-value)
p-tau217 [pg/mL], median (IQR)	0.19 (0.08–0.28)	0.11 (0.07–0.14)	0.43 (0.25–0.61)	0.65 (0.38–0.9)	**<0.001** (model p-value)

Bold p-valus indicate statistical significance (p<0.05)

Age, sex, and biomarker data (p-tau181, p-tau217) are presented as median (IQR), or N (%), as appropriate. Group comparisons were performed using age- and sex-adjusted multiple linear regression. *P*-values indicate model *p*-value

*AD* Alzheimer’s disease, *ALS* amyotrophic lateral sclerosis, *IQR* interquartile range, N number of cases, *SMA* spinal muscular atrophy

SMA patients were younger than ALS and AD patients, with a significant overall age difference between groups (Table 1). This was expected, given the naturally differing ages of onset of the diseases. No age difference was observed between SMA and disease controls, and sex distribution did not differ significantly between groups. The higher proportion of males in the ALS group is consistent with the known sex prevalence. BMI did not differ relevantly between groups in the subset with available data (Table S15).

Baseline and follow-up characteristics of the SMA cohort are summarised in Table 2 (additional details in Table S7). Mean disease duration at baseline was 30.4 years. FUP1 and FUP2 corresponded to months 9–16 and 22–26 of treatment, respectively. Clinical characteristics did not differ between cohorts at baseline.

**Table 2:** Demographic and clinical characteristics of the SMA cohort

	Overall SMA	Halle	Ulm	Leuven
*n*	70	7	29	34
Baseline	65	2	29	34
Follow-up 1	40	6	0	34
Follow-up 2	63	2	28	33
Age [years], median (IQR)				
Baseline	35.00 (24.00–46.50)	49.50 (43.00–56.00)	30.00 (18.00–46.00)	35.50 (26.00–48.75)
Follow-up 1	36.50 (27.00–51.25)	38.50 (21.25–57.75)	–	36.50 (27.00–49.75)
Follow-up 2	37.60 (26.00- 50.00)	59.5 (58.00- 61.00)	33.00 (21.00- 49.00)	37.00 (27.50- 46.50)
Female, *n* (%)	31 (44.28)	2 (28.57)	11 (37.93)	18 (52.94)
SMA type, *n* (%)				
I	3 (4.29)	0	3 (10.35)	0
II	20 (28.17)	2 (28.57)	9 (31.03)	9 (26.47)
III	43 (61.43)	4 (57.14)	17 (58.62)	22 (64.71)
IV	5 (7.14)	1 (14.29)	0	4 (11.76)
SMN2 copy number, *n* (%)				
2	5 (7.14)	0	3 (10.35)	2 (5.88)
3	48 (68.57)	1 (14.29)	21 (72.4)	26 (76.47)
4	13 (18.57)	2 (28.57)	5 (17.2)	6 (17.64)
Unknown	4 (5.71)	4 (57.14)	0	0
Therapy, *n* (%)				
Risdiplam	25 (35.71)	6 (85.71)	1 (3.45)	18 (52.94)
Nusinersen	45 (64.29)	1 (14.29)	28 (96.55)	16 (47.06)
Disease Duration [years], (mean ± SD)				
Baseline	30.42 (± 13.39)	39.00 (± 19.80)	26.95 (± 14.05)	32.88 (± 12.22)
Follow-up 1	33.85 (± 32.20)	33.67 (± 13.25)	–	33.88 (± 12.22)
Follow-up 2	32.20 (± 13.06)	44.00 (± 15.56)	26.56 (± 14.05)	33.73 (± 11.80)
HFMSE%, (mean ± SD)^a^				
Baseline	33.26 (± 31.85)	58.33 (± 35.36)	26.34 (± 31.28)	45.23 (± 30.61)
Follow-up 1	45.61 (± 33.91)	50.61 (± 44.89)	–	43.94 (± 31.19)
Follow-up 2	34.63 (± 32.94)	86.36 (± 0.00)	27.82 (± 32.12)	42.53 (± 31.49)
RULM%, (mean ± SD)				
Baseline	54.23 (± 31.86)	100.00 (± 0)	48.32 (± 31.06)	55.94 (± 31.41)
Follow-up 1	61.89 (± 31.68)	76.76 (± 30.91)	–	59.70 (± 31.68)
Follow-up 2	58.20 (± 30.74)	100.00 (± 0)	53.60 (± 30.22)	60.28 (± 30.85)
MFM32%, (mean ± SD)^b^				
Baseline	–	–	–	28.53 (± 24.09)
Follow-up 1	–	–	–	30.85 (± 24.47)
Follow-up 2	–	–	–	32.23 (± 23)
FVC [% predicted], (mean ± SD)				
Baseline	67.65 (± 31.10)	86.00 (± 2.83)	59.67 (± 33.59)	73.06 (± 28.67)
Follow-up 1	81.04 (± 30.27)	97.25 (± 25.89)	–	75.15 (± 29.83)
Follow-up 2	71.71 (± 31.93)	108.30 (± 18.76)	58.44 (± 29.57)	74.09 (± 29.61)

Age is presented as median and interquartile range (IQR), sex, and clinical characteristics (SMA type, SMN2 copy number, treatment, disease duration, HFMSE, RULM, and FVC % predicted) are presented as mean ± standard deviation (SD) or as absolute numbers and percentages (n, %). RULM and HFMSE scores are reported as percentages; absolute values are provided in the Supplementary Material Table S7. a HFMSE was collected only in nusinersen-treated patients within the Leuven cohort. b MFM32 was available exclusively in risdiplam-treated patients and was collected only at the Leuven center

*FVC *forced vital capacity, *HFMSE* Hammersmith functional motor scale–expanded, *IQR* interquartile range, *MFM32* Motor function measure 32, *RULM* Revised upper limb module, *SD* standard deviation

### Concentrations of blood p-tau181 and p-tau217 in different diagnostic groups at baseline

**Fig. 1:**
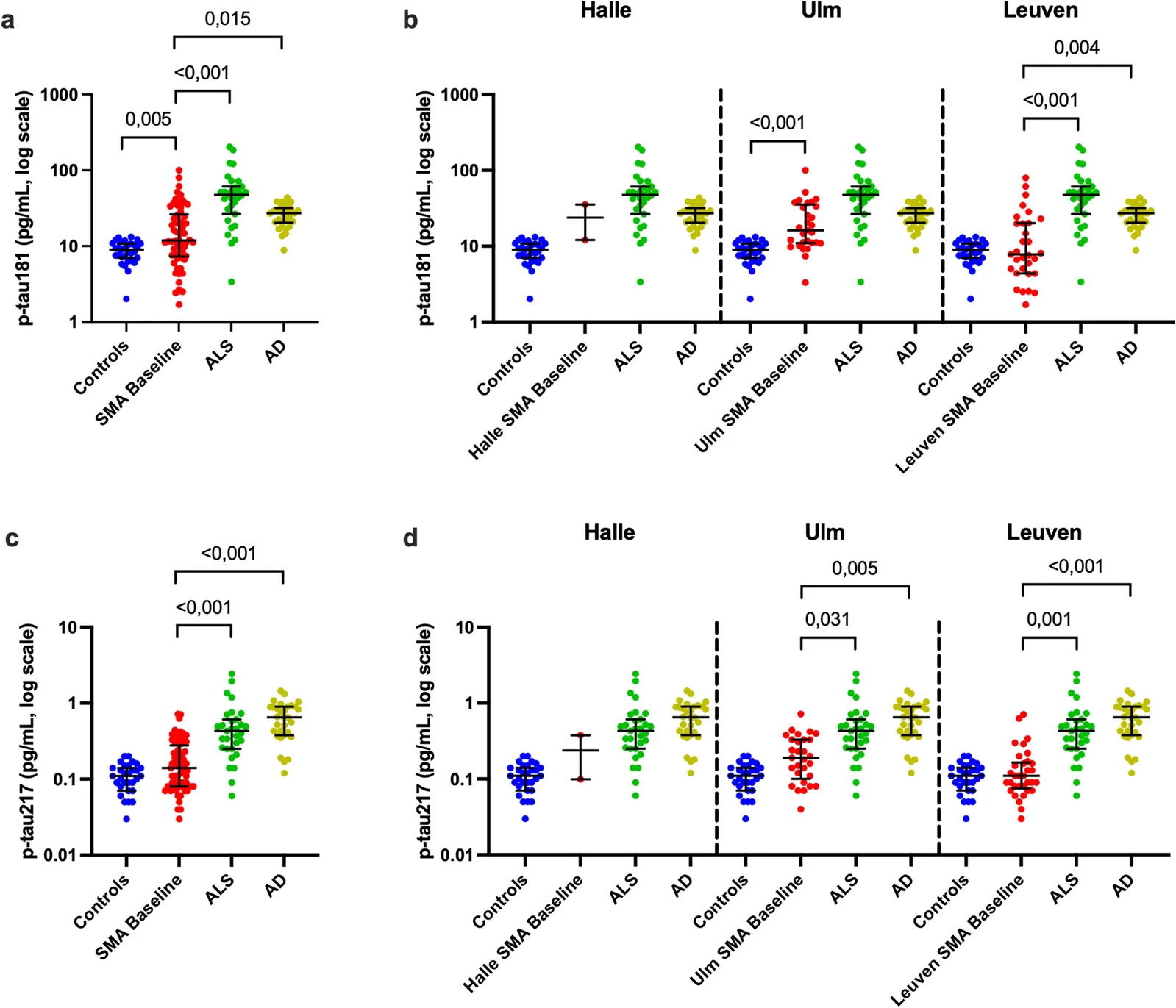
Serum p-tau181 and p-tau217 levels in SMA, Controls, ALS, and AD. Plots depict the distribution of biomarker levels in patients with SMA, Controls, ALS, and AD, with medians and interquartile ranges indicated; individual data points are overlaid. Group differences between SMA, Controls, ALS, and AD were assessed using multiple linear regression models, with diagnostic group included as a categorical predictor and age and sex as covariates. SMA was used as the reference group. Pairwise comparisons refer to age- and sex-adjusted differences relative to SMA. **a** p-tau181 in non-neurodegenerative Controls (*n *= 31), SMA (*n *= 62), ALS (*n *= 31), and AD (*n *= 31). **b** p-tau181 in the Halle (*n *= 2), Ulm (*n *= 29) and Leuven (*n *= 31) SMA baseline cohort in comparison to non-neurodegenerative Controls, SMA Baseline, ALS and AD. **c** p-tau217 in non-neurodegenerative Controls (*n *= 31), SMA (*n *= 64), ALS (*n *= 31), and AD (*n *= 31). **d** p-tau217 in the Halle (*n *= 2), Ulm (*n *= 29) and Leuven (*n *= 33) SMA baseline cohort in comparison to non-neurodegenerative Controls, SMA Baseline, ALS and AD. No statistical testing was performed for the Halle cohort due to the small sample size. The *y*-axis is displayed on a logarithmic (base–10) scale. *AD* Alzheimer’s disease, *ALS* Amyotrophic lateral sclerosis, *SMA* Spinal muscular atrophy

After adjustment for age and sex using multiple linear regression, p-tau181 levels differed across diagnostic groups (model *p *< 0.001). Compared with SMA, levels were lower in disease controls (*β *= −0.48, 95%-CI −0.81 to −0.15) and higher in ALS (*β *= 0.99, 95% -CI 0.58 to 1.39) and AD (*β *= 0.56, 95%-CI 0.11 to 1.00) (Fig. 1a; Table S1).

For p-tau217 (model *p*<0.001), no difference was observed between SMA and controls (*β *= −0.09, 95%-CI −0.22 to 0.05), whereas levels were higher in both ALS (*β *= 0.32, 95%-CI 0.16 to 0.49) and AD (*β *= 0.45, 95%-CI 0.26 to 0.63) compared with SMA (Fig. 1c; Table S1).

In centre-stratified analyses, p-tau181 in the Ulm cohort (*n *= 29) was lower in disease controls compared with SMA (*β *= −0.52, 95%-CI −0.73 to −0.31), with no difference between SMA and ALS (*β *= 0.13, 95%-CI −0.14 to 0.39) or AD (*β *= 0.21, 95%-CI −0.08 to 0.49) (Fig. 1b; Table S3). In the Leuven cohort (*n *= 31), no difference between SMA and controls was detected despite substantial sample variability (*β *= −0.20, 95%-CI −0.56 to 0.17), whereas levels were higher in ALS (*β *= 1.22, 95%-CI 0.77 to 1.67) and AD (*β *= 0.73, 95%-CI 0.24 to 1.22) compared with SMA (Fig. 1b; Table S4).

For p-tau217, no relevant SMA–control difference was observed in either the Ulm (*β *= −0.14, 95%-CI −0.32 to 0.04) or Leuven cohort (*β *= −0.06, 95%-CI −0.22 to 0.11). In the Ulm cohort (n=29), levels were higher in ALS (*β *= 0.25, 95%-CI 0.02 to 0.48) and AD (*β *= 0.37, 95%-CI 0.11 to 0.62) compared with SMA (Fig. 1d; Table S3). In the Leuven cohort (*n *= 33), levels were similarly higher in ALS (*β *= 0.35, 95%-CI 0.14 to 0.56) and AD (*β *= 0.47, 95%-CI 0.24 to 0.70) relative to SMA (Fig. 1d; Table S4).

Due to the limited sample size of the Halle cohort (*n* = 2 at baseline), no statistical testing was performed.

### p-tau levels at different time points

**Fig. 2:**
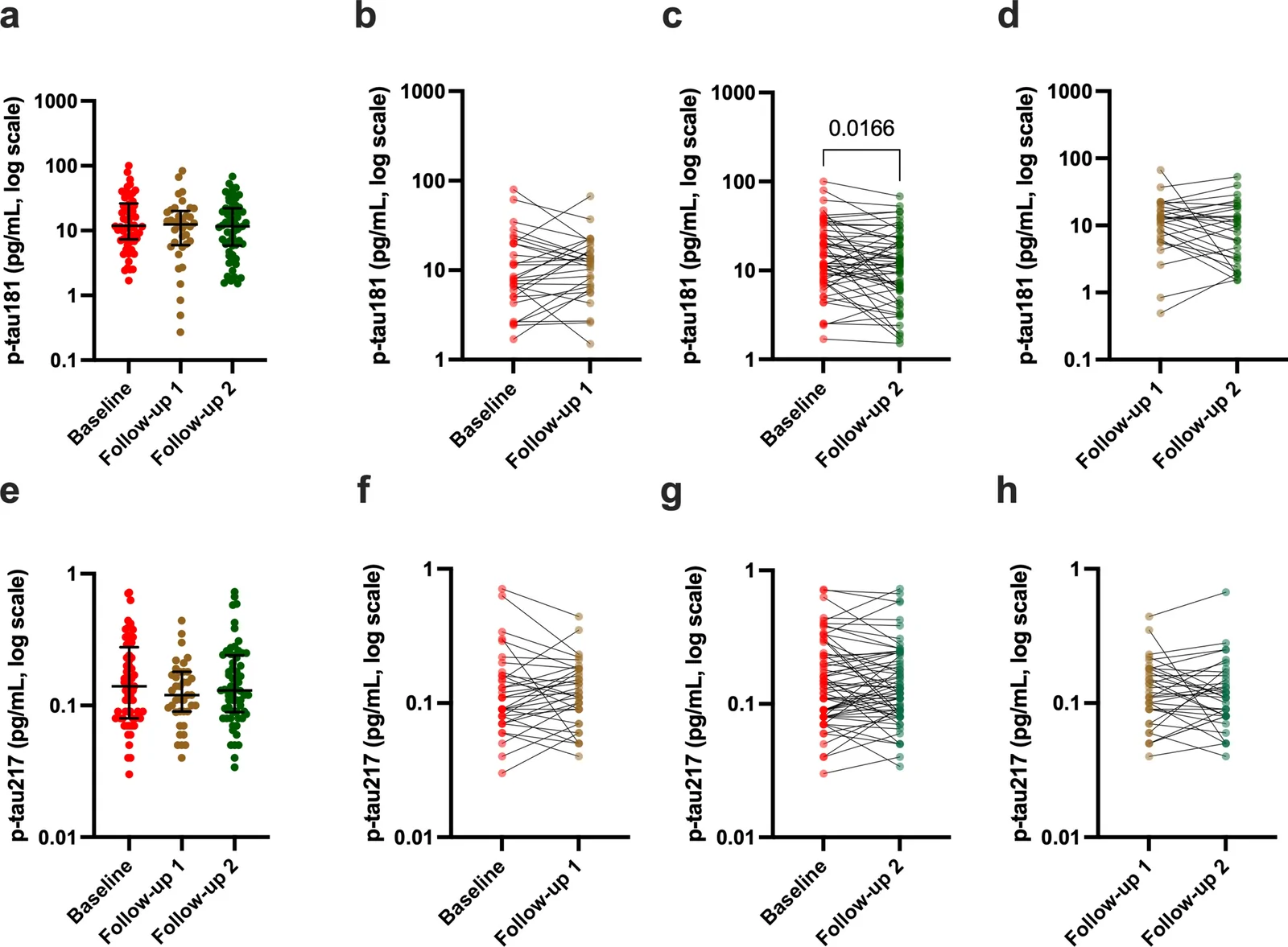
p-tau181 and p-tau217 in the SMA cohort at different time points. **a** p-tau181 at baseline (*n *= 62), follow-up 1 (*n *= 37) and follow-up 2 (*n *= 59). **e** p-tau217 at baseline (*n *= 64), follow-up 1 (*n *= 40) and follow-up 2 (*n *= 63). Plots depict the distribution of biomarker levels in patients at different time points, with medians and interquartile ranges indicated; individual data points are overlaid. Group comparisons across time points were performed using the Kruskal–Wallis-test based on available data per time point with Dunn’s multiple comparison adjustment. No statistical difference was found. **b, c, d, f, g, h** Paired comparisons of p-tau181 and p-tau217 levels between baseline and follow-up 1 (**b** and **f**, p-tau181 *n* = 29, p-tau217 *n *= 35), baseline and follow-up 2 (**c** and **g**, p-tau181 *n *= 56, p-tau217 *n *= 62), and follow-up 1 and follow-up 2 (**d** and **h**, p-tau181 *n *= 28, p-tau217 *n *= 34) in patients with available data at both time points. Lines connect paired observations. Statistical comparisons were performed using the Wilcoxon signed-rank test. Due to incomplete longitudinal data, analyses were conducted using available-case data and should be considered exploratory. The *y*-axis is displayed on a logarithmic (base–10) scale

In an unpaired analysis (Kruskal–Wallis), p-tau181 and p-tau217 levels did not differ relevantly between baseline, FUP1, and FUP2 in the overall SMA cohort (Fig. 2a, e; Table S5). In paired Wilcoxon signed-rank analysis limited to patients with measurements at both time points (Fig. 2, Table S6), p-tau181 levels were lower at FUP2 compared with baseline (Δ median −1.80 pg/mL, IQR −5.90 to 2.10, *p *= 0.017; *n *= 56), suggesting a within-patient reduction (Fig. 2c). No relevant change was observed between baseline and FUP1 (Δ 0.17 pg/mL, IQR −7.90 to 5.10; *n *= 29) or between FUP1 and FUP2 (Δ 0.00 pg/mL, IQR −5.90 to 5.70; *n *= 28) with limited data points being available (Fig. 2b, d). P-tau217 showed no relevant change at any time point (Δ range 0.00 to −0.01 pg/mL; Fig. 2f–h; Table S6).

In an exploratory cross-sectional analysis at FUP3 (4–5 years; *n *= 8), p-tau181 and p-tau217 values were within the upper range of those observed at earlier time points, without clear directional change. Given the very limited sample size, no statistical testing or clinical correlation was performed, and these findings should be interpreted with caution (Figure S1; Tables S13–14). Paired analysis was not possible as no baseline data were available for these patients.

### p-tau in correlation with clinical data, neurofilaments and creatine kinase

**Fig. 3:**
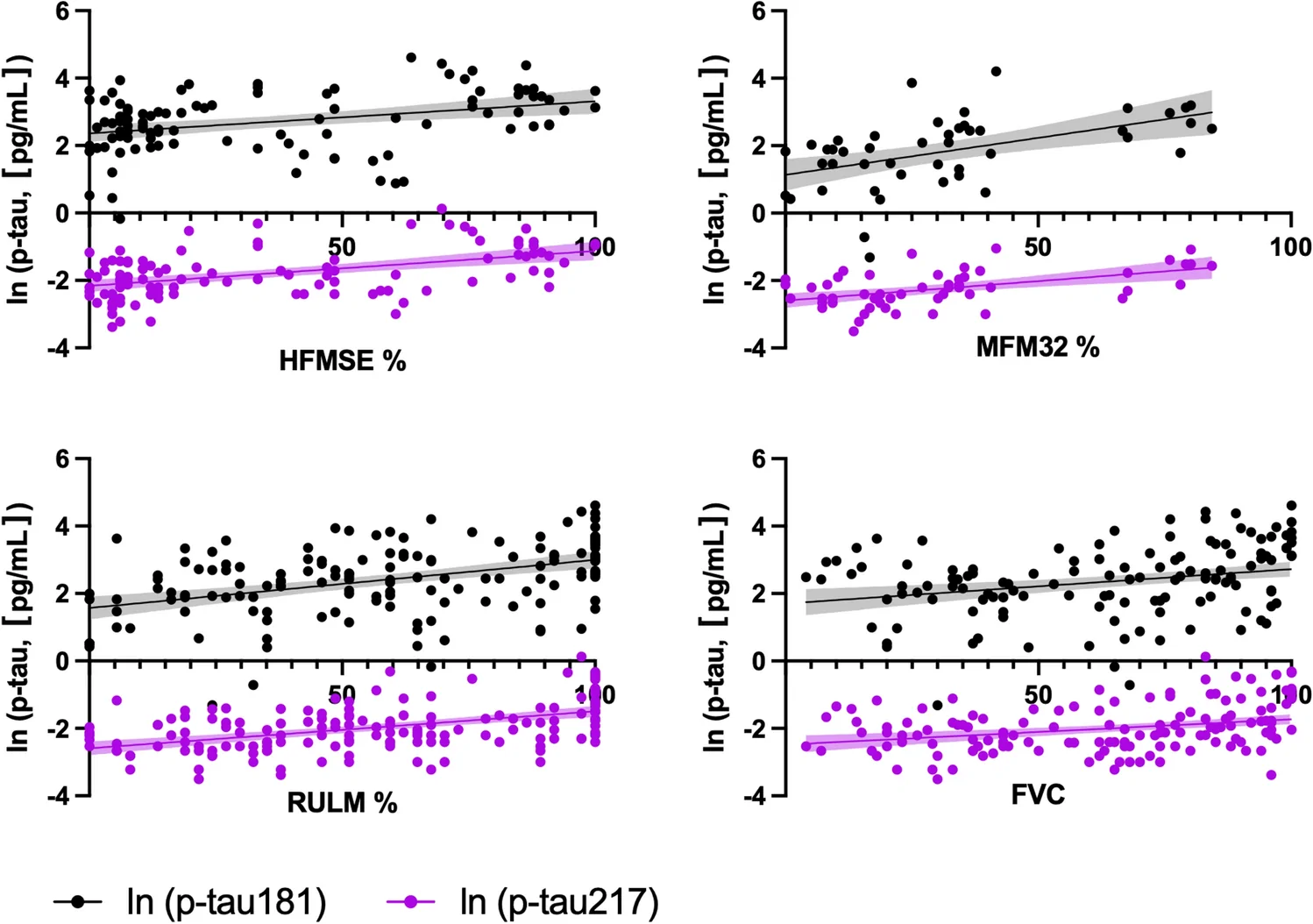
Associations of p-tau181 and p-tau217 with motor and respiratory outcomes in SMA*. *Scatterplots showing the association between ln-transformed p-tau181 and 217 and HFMSE %, RULM %, MFM32%, FVC. The regression line and 95% confidence band are shown for visualization purposes only; statistical inference was based on linear mixed-effects models accounting for repeated measurements. *FVC* forced vital capacity, *HFMSE* Hammersmith functional motor scale–expanded, *MFM32* Motor function measure 32, *RULM* Revised upper limb module

**Fig. 4:**
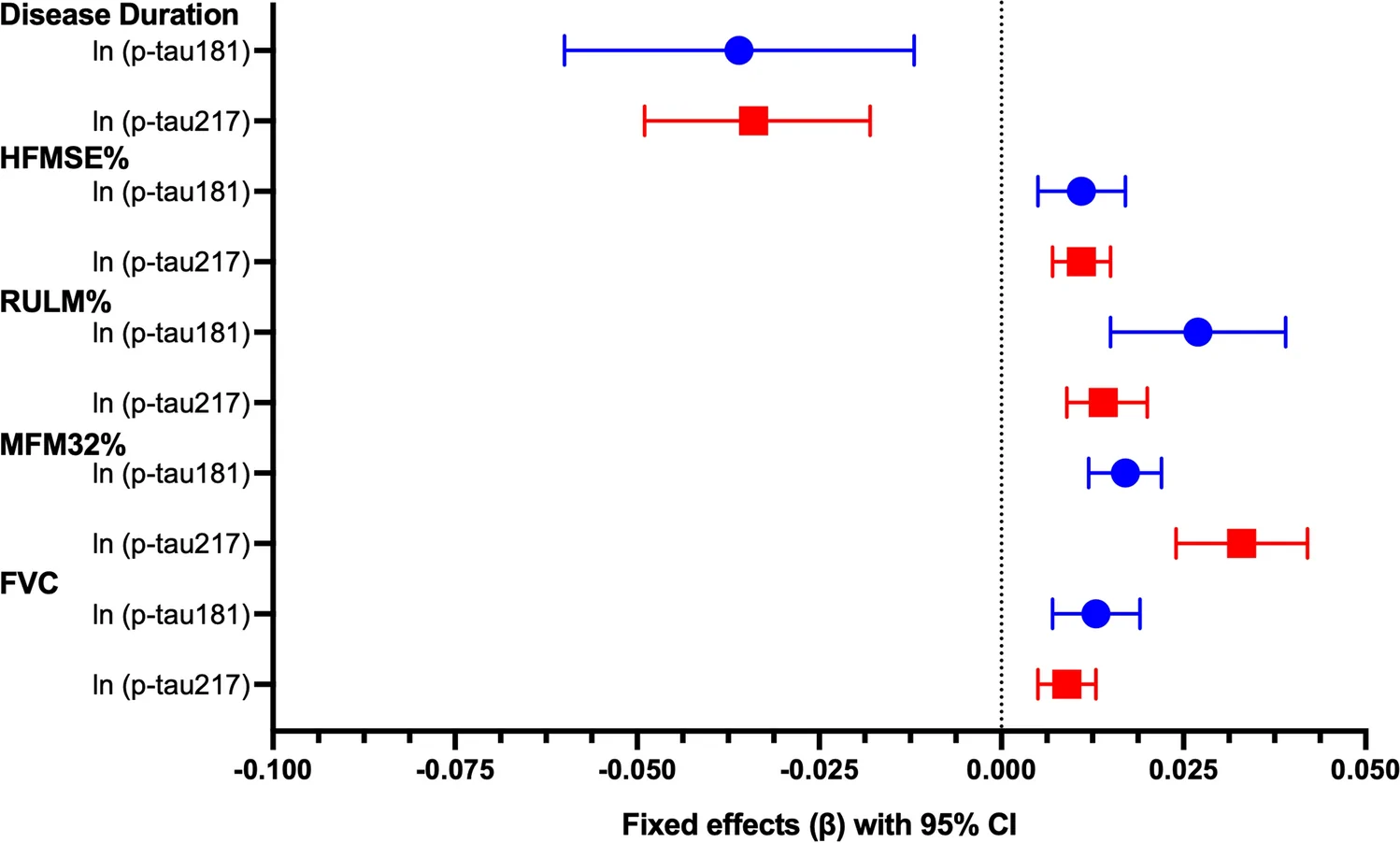
Forest plot of associations between continuous clinical predictors and ln-transformed serum p-tau levels in SMA. Forest plot showing β coefficients with 95% confidence intervals derived from linear mixed-effects models examining associations between continuous predictors (Disease duration, HFMSE%, RULM%, MFM32%, FVC) and ln-transformed plasma p-tau181 and p-tau217 levels. Linear mixed-effects models with random intercept for PatientID, adjusted for timepoint, center, age, and sex. Effect estimates represent the change in ln(p-tau) per one-unit increase in the respective predictor. The vertical reference line indicates no association (*β* = 0). *P*-values were adjusted for multiple testing using false discovery rate (FDR) correction. MFM32 data were available only for patients treated with risdiplam and were collected exclusively at the Leuven center. *CI* confidence interval, *FVC* forced vital capacity, *HFMSE* Hammersmith Functional Motor Scale–Expanded, *MFM*32 Motor Function Measure 32, *RULM* Revised Upper Limb Module

Higher motor function scores were consistently associated with higher levels of both serum p-tau181 and p-tau217. Significant positive associations were observed for HFMSE, HFMSE%, RULM, RULM%, and FVC (all FDR-adjusted *p*<0.001; Table 3, S9; Fig. 3, 4). In the Leuven cohort, MFM32 total and domain scores were positively associated with both p-tau181 and p-tau217 levels. Disease duration was inversely associated with both biomarkers, indicating lower levels with longer disease duration (p-tau181: *p *= 0.010; p-tau217: *p*<0.001).

**Table 3:** Linear mixed-effects models of serum p-tau levels and continuous clinical measures, neurofilament light chain and creatine kinase in SMA

Predictor	Outcome	*β* (Estimate)	95% CI	*p*-value (FDR-adj.)	*n*
Continuous clinical variables, neurofilament light chain and creatine kinase					
HFMSE%	p-tau181 (ln)	0.01	0.01 to 0.02	**0.001**	101
	p-tau217 (ln)	0.01	0.01 to 0.02	**<0.001**	102
RULM%	p-tau181 (ln)	0.02	0.01 to 0.02	**<0.001**	147
	p-tau217 (ln)	0.01	0.01 to 0.02	**<0.001**	156
MFM32%	p-tau181 (ln)	0.03	0.02 to 0.04	**0.006**	46
	p-tau217 (ln)	0.01	0.01 to 0.02	**<0.001**	53
FVC (% predicted)	p-tau181 (ln)	0.01	0.01 to 0.02	**<0.001**	150
	p-tau217 (ln)	0.01	0.01 to 0.01	**<0.001**	158
Disease Duration	p-tau181 (ln)	0.04	−0.06 to − 0.01	**0.010**	158
	p-tau217 (ln)	0.03	−0.05 to − 0.02	**<0.001**	167
CK (ln)	p-tau181 (ln)	0.64	0.47 to 0.81	**<0.001**	98
	p-tau217 (ln)	0.38	0.28 to 0.48	**<0.001**	107
NfL (ln)	p-tau181 (ln)	0.27	−0.52 to –0.03	0.063	113
	p-tau217 (ln)	0.05	−0.23 to 0.13	0.623	115

Bold p-values indicate statistical significance after FDR adjustment (adjusted p<0.05)

Linear mixed-effects models with a random intercept for PatientID. adjusted for time point, center, age, and sex. Estimates represent β coefficients for ln-transformed outcomes. *P*-values were adjusted using the false discovery rate (FDR). RULM and HFMSE scores are reported as percentages; absolute values are provided in the Supplementary Material. MFM32 data were available only for patients treated with risdiplam and were collected exclusively at the Leuven center

*CI* confidence interval, *CK* creatine kinase, *FDR* false discovery rate, *FVC* forced vital capacity, *HFMSE* Hammersmith functional motor scale–expanded, *MFM32* Motor function measure 32, *NfL* Neurofilament light chain, *RULM* Revised upper limb module

Motor function scores remained stable between baseline and FUP2, with no relevant change in HFMSE or RULM (Table 2, S7).

In exploratory multivariable linear regression analyses (Table S11), higher baseline p-tau181 was associated with greater HFMSE decline (*β *= 1.79, 95%-CI 0.59 to 2.99), whereas the association with p-tau217 was smaller and less certain (*β *= 1.60, 95%-CI −0.13 to 3.33). Age, sex, and baseline HFMSE did not relevantly contribute to HFMSE changes. These findings should be seen as preliminary.

For ΔRULM, neither biomarker showed a relevant association (p-tau181: *β *= 0.48, 95%-CI −0.35 to 1.30; p-tau217: *β *= 0.48, 95%-CI −0.66 to 1.62). Instead, male sex (*β *= 1.82, 95%-CI 0.48 to 3.16; *β *= 1.83, 95%-CI 0.46 to 3.20) and older age (*β *= −0.06, 95%-CI −0.10 to −0.01; *β *= −0.06, 95%-CI −0.11 to −0.01) were the primary determinants of RULM change (Table S11).

Global likelihood ratio tests revealed a significant overall association between SMA type and both p-tau181 and p-tau217 levels (FDR-adjusted *p*<0.001; Table 4). However, linear mixed effects models between SMA types remained statistically non-significant after FDR correction (Table S12). Type 4 showed higher levels compared to type 1 (p-tau181: *β *= 1.15, 95%-CI 0.00 to 2.30; p-tau217: *β *= 0.90, 95%-CI 0.09 to 1.71), but these associations did not withstand multiple testing correction. No significant differences were observed between SMA types 1 vs. 2 or 1 vs. 3 for either biomarker.

**Table 4:** Global likelihood ratio tests for associations between categorical clinical variables and serum p-tau levels in SMA

Predictor	Outcome	*χ*^2^	df	*p*-value (FDR-adj.)	*n*
SMA type	p-tau181 (ln)	21.08	3	**<0.001**	167
	p-tau217 (ln)	24.61	3	**<0.001**	158
SMN2 copy number	p-tau181 (ln)	2.82	3	0.448	163
	p-tau217 (ln)	2.81	3	0.448	154
Therapy	p-tau181 (ln)	0.56	1	0.469	167
	p-tau217 (ln)	2.48	1	0.136	158
Therapy stage	p-tau181 (ln)	9.22	8	0.367	167
	p-tau217 (ln)	13.57	8	0.114	158
Functional status	p-tau181 (ln)	34.03	2	**<0.001**	110
	p-tau217 (ln)	27.51	2	**<0.001**	102

Bold p-values indicate statistical significance after FDR adjustment (adjustment p<0.05)

Global likelihood ratio tests from linear mixed-effects models assessing associations between categorical clinical variables and ln-transformed p-tau181 and p-tau217 levels, adjusted for time point, centre, age, and sex. *P*-values were corrected for multiple testing using the false discovery rate (FDR)

*df* degrees of freedom, *FDR* false discovery rate, *χ*^2^ likelihood ratio chi-square

Functional status showed a significant overall association with both biomarkers (FDR-adjusted *p*<0.001; Table 4). Both ambulatory patients (p-tau181: *β *= 1.33, 95%-CI 0.82 to 1.85; p-tau217: *β *= 0.93, 95%-CI 0.65 to 1.21) and those ambulatory with walking aids (p-tau181: *β *= 1.56, 95%-CI 0.76 to 2.37; p-tau217: *β *= 0.66, 95%-CI 0.21 to 1.11) had higher levels compared with non-ambulatory individuals (Table S12). No significant global effects were observed for SMN2 copy number or therapy after FDR-adjustment. Given the absence of relevant differences between nusinersen and risdiplam, no additional subgroup analyses were performed.

CK was positively associated with both p-tau181 and p-tau217 (all FDR-adjusted *p*-values <0.001), while no significant association was observed for NfL after FDR correction (Table 3, S10; Figure S2).

NfL differed across time points (*p *= 0.030), decreasing from baseline to follow-up 2 (Table 3, S10; Figure S2-S3). Dunn’s post-hoc test showed a significant difference between baseline and follow-up 2 (*p *= 0.045, Table S16).

## Discussion

We investigated serum p-tau181 and p-tau217 levels longitudinally and in association with clinical data in adult 5q SMA, providing initial insights into their potential as biomarkers.

Consistent with a previous preliminary study in treatment-naïve SMA, serum p-tau181 was elevated in SMA compared with disease controls at baseline [18]. In contrast to Vacchiano et al., p-tau181 levels in our cohort were lower than in AD, which may in part reflect differences in disease progression, as the earlier study included a higher proportion of rapidly progressive SMA type II patients. With the rapid decline leading to higher p-tau levels, potentially comparable to ALS which showed no difference to AD in previous studies [16]. In our study p-tau levels in SMA were also lower than those observed in ALS, which might equally be due to the less rapid decline in our more adult SMA population.

Using within-subject comparisons, we observed a significant decline in p-tau181 levels between baseline and FUP2, with no consistent change at other time points. This reduction may reflect slow ongoing disease progression in adult SMA patients under treatment, potentially associated with reduced release of p-tau at more advanced disease stages and lower muscle volume. This interpretation is in line with our cross-sectional findings of lower p-tau levels in patients with reduced motor function and poorer functional status.

The lack of a longitudinal change in p-tau217 is consistent with our baseline findings, in which p-tau217 did not differ between SMA and disease controls. In a recent study p-tau217 in ALS showed a strong association with the disease severity whereas p-tau181 correlated more strongly with lower motoneuron damage, as previously shown in ALS cohorts [16, 24]. This may support our finding of elevated p-tau181 in SMA, given that SMA is characterized by isolated lower motor neuron involvement. Therefore, p-tau181 and p-tau217 may capture different aspects of motor neuron and muscle pathology, while the precise pathophysiological basis remains to be elucidated.

Overall, these findings should be interpreted cautiously. The cohort had a long mean disease duration at baseline and was followed over a comparatively short period, potentially limiting the detection of meaningful change in a slowly progressive disease. This is further supported by the lack of significant changes in motor scores over time. The limited sample size at FUP1 further constrains the paired analyses at that time point.

To contextualise these findings clinically, we assessed associations between p-tau levels and clinical parameters. Consistent with the longitudinal findings, serum p-tau181 and p-tau217 levels were positively associated with motor function across multiple scales and with preserved respiratory function, which indicate more muscle mass at an earlier disease state. This was also supported by higher p-tau levels linked to shorter disease duration and higher functional status. Furthermore, a strong positive association between creatine kinase (CK) and p-tau was observed. CK has been previously shown to correlate positively with motorscores and higher functional skills in SMA patients under treatment [25]. This supports our findings of higher motoric skills and, therefore, higher CK being positively associated with p-tau. These findings suggest that peripheral p-tau might reflect active denervation or muscle-related remodeling rather than irreversible neuronal or muscular damage.

There was no association between p-tau peptides and neurofilament light chains (NfL), reinforcing evidence that p-tau biomarkers reflect peripheral rather than neuroaxonal damage in SMA and that NfL may have limited utility in adult SMA [7, 26, 27]. The observed decline in NfL under therapy aligns with a recent study reporting a slight treatment-associated decrease, though findings in adult SMA remain inconsistent [28].

To our knowledge, this is the first study to report associations between p-tau and clinical parameters in adult SMA, limiting direct comparison with existing literature. Available data derive predominantly from paediatric SMA and ALS, both more rapidly progressive motor neuron diseases.

In ALS, peripheral p-tau181 levels increase over time, and higher levels are associated with faster clinical decline and lower motor function in its natural disease course [16–18]. In paediatric SMA, CSF p-tau reductions under treatment have been reported, likely reflecting treatment response rather than natural disease course [29, 30].

The dynamics of p-tau release likely differ between rapidly and slowly progressive motor neuron diseases. In ALS and paediatric SMA, muscle atrophy occurs quickly and extensively, often affecting vital functions early. In this context, p-tau levels are elevated and may continue to rise over time, reflecting the sudden and widespread degeneration of muscle tissue [16, 18].

In contrast, adult SMA typically progresses more slowly. Here, muscle loss is gradual, and p-tau may initially be higher as muscle fibres are slowly released and undergo degeneration. As the disease advances and the total muscle mass declines, less p-tau can be released, leading to stable or even declining serum levels over time.

These observations suggest that both patterns, rising p-tau in rapid-onset disease and plateauing or declining p-tau in slowly progressive disease, can coexist, reflecting different pathophysiological processes.

Building on these findings, we assessed whether baseline p-tau levels predicted functional decline. While overall regression models for ΔHFMSE did not reach significance, higher baseline p-tau181 was associated with greater HFMSE decline, whereas p-tau217 showed a non-significant trend. Age, sex, and baseline motor function had minimal impact, suggesting that p-tau181 may provide specific predictive information for functional deterioration as captured by HFMSE. Given the exploratory nature of this analysis, this finding should be considered hypothesis-generating and requires confirmation in future studies before p-tau can be considered for prognostic use. No relevant association was observed for ΔRULM, potentially reflecting ceiling effects in adult SMA [31].

Strengths of this study include the multi-centre longitudinal design, relatively large sample size, comprehensive clinical phenotyping, and use of linear mixed-effects models with appropriate covariate adjustment. Limitations include small subgroup sizes, few time points, and advanced disease stage at baseline. Motor scores exhibit floor and ceiling effects, with RULM ceiling effects particularly relevant in adult SMA [31]. BMI could not be included as a covariate due to missing data; though exploratory analyses did not suggest a relevant influence on p-tau levels. Furthermore, we did not analyse p-tau peptides in CSF and all analyses were performed under treatment, with therapeutic effects in slowly progressive adult SMA remaining incompletely understood.

Future studies should evaluate serum p-tau at earlier disease stages in adult SMA and in paediatric SMA, where disease dynamics may more closely resemble ALS. Integration of imaging or electrophysiological measures may further clarify the biological substrate of peripheral p-tau elevations. Paired CSF-serum sampling in future studies would allow a more direct test of the peripheral-versus-central origin of circulating p-tau in SMA, analogous to the serum-CSF dissociation already described in ALS [16].

These findings also carry practical implications for the clinical interpretation of circulating p-tau, which is increasingly used as a blood-based screening and diagnostic tool for AD [32, 33]. Our data indicate that motoneuron diseases are associated with serum p-tau elevation independently from AD pathology. In clinical settings where p-tau is used to support an AD diagnosis or to triage patients for further testing, comorbid motoneuron diseases should be considered a potential confounder, and elevated peripheral p-tau in such patients should not be over-interpreted as evidence of concomitant AD-type pathology without corroborating CSF AD biomarkers biomarkers.

In conclusion, high p-tau may reflect active, widespread muscle degeneration in rapidly progressive conditions, whereas in slowly progressive adolescent and adult SMA, serum p-tau appears to mirror ongoing muscle turnover early in the disease, but declines as total muscle tissue decreases. In this context, peripheral p-tau181 may serve as a biomarker of active denervation. Its association with motor performance suggests potential utility for monitoring disease progression and for identifying patients at risk of functional deterioration. Further longitudinal studies are warranted to validate p-tau adding clinical value and as a predictive marker and to explore its integration into clinical assessment and therapeutic decision-making in adult SMA.

## Supplementary Information

Below is the link to the electronic supplementary material.

Supplementary file1 (DOCX 227 KB)

## Data Availability

All data used for this study is available on reasonable request by contacting the authors.
