# Supplementary material for: Serum p-tau181 and p-tau217 levels in adolescents and adults with 5q spinal muscular atrophy
Source: J Neurol. 2026 Oct 21;273(11):672. doi: 10.1007/s00415-026-14194-1 (PMC13687299; doi:10.1007/s00415-026-14194-1)
Supplement: Supplementary file 1 [file 415_2026_14194_MOESM1_ESM.docx]

**Supplementary Information**

**SUPPLEMENTARY METHODS**

Biomarker analyses

The p-tau181 and p-tau217 assays were commercially obtained from Quanterix and performed on the HD-X Analyzer.

All SMA samples were analyzed using the *p-Tau181 Advantage PLUS Kit*, which has been validated by the manufacturer for use with serum. Measurements were conducted according to the manufacturer’s recommendations, using a 1:4 dilution and a sample volume of 55 µL. Disease control, Alzheimer’s disease (AD), and amyotrophic lateral sclerosis (ALS) samples were quantified using the *pTau181-Advantage V2 Kit*, with results converted to pTau181-Advantage V2.1 values as specified by the manufacturer. Equivalence between the p-Tau181 Advantage PLUS Kit and the pTau181-Advantage V2.1 Kit has been demonstrated by the manufacturer. All included values were above the limit of detection (LOD: 0.724pg/ml).

All samples for p-tau217 were analyzed using the *ALZpath p-Tau217 Advantage PLUS Kit*. This kit has been primarily validated by the manufacturer for use with plasma. However, multiple studies have demonstrated a strong correlation between serum and plasma p-tau217 concentrations, indicating that the kit can reliably quantify p-tau217 in serum samples [1]. A sample volume of 63 µL with a 1:3 dilution was used according to the manufacturer’s recommendations. All included values were above the limit of detection (LOD: 0.0008pg/ml).

Neurofilament light chain was measured using the commercially available *Simple Plex™ NF-L Assay* on Ella™ Kit from biotechne.

Linear mixed-effects models

Linear mixed-effects models (LMMs) were applied to investigate longitudinal associations between blood-based biomarkers and clinical parameters while accounting for repeated measurements within individuals. Log-transformed p-tau181 and p-tau217 concentrations were used as dependent variables to reduce skewness and approximate normality of residuals. All models included timepoint (baseline, follow-up 1, follow-up 2), age, and sex as fixed effects. Study center was included as an additional fixed effect where applicable. A random intercept for patient was included in all models to account for within-subject correlations. Models were fitted using maximum likelihood estimation as implemented in the lme4 package, with inference for fixed effects based on Satterthwaite’s approximation (lmerTest package).

For continuous clinical variables, separate LMMs were fitted for each predictor. Model estimates are reported as regression coefficients (β) with corresponding standard errors and 95% confidence intervals, representing the expected change in log-transformed biomarker concentration per unit increase in the predictor. Continuous predictors included disease duration, functional scores (HFMSE, HFMSEpro, RULM, RULMpro, MFM32 and its domain subscores), vital capacity (VC), and log-transformed biochemical markers (CK and NfL). Statistical significance of continuous predictors was assessed using Wald t-tests, and p-values were adjusted for multiple testing using the Benjamini–Hochberg false discovery rate (FDR) procedure.

Categorical clinical variables were analyzed using LMMs including the respective factor as a fixed effect. For these models, overall (global) effects of the categorical predictors were evaluated using likelihood-ratio tests, comparing full models including the predictor of interest with reduced models excluding that predictor while retaining all covariates and the random-effects structure. Categorical predictors included SMA type, SMN2 copy number, therapy status, therapy stage, and functional status. Results are reported as likelihood-ratio chi-square statistics (χ²), degrees of freedom, and corresponding p-values. Multiple testing across categorical predictors was controlled using the Benjamini–Hochberg FDR procedure.

Model specification in R:

lmer(outcome ~ predictor + timepoint + age + sex + center + (1|patient))

**SUPPLEMENTARY TABLES**

**Supplementary Table S1:** **Baseline p-tau levels across diagnostic groups**

| Biomarker | Model p-value | Group | β (Estimate) | 95% CI | p-value |
| --- | --- | --- | --- | --- | --- |
| p-tau181 (ln) | **<0.001** | SMA vs. Controls | -0.48 | -0.81 to -0.15 | **0.005** |
|  |  | SMA vs. ALS | 0.99 | 0.58 to 1.39 | **<0.001** |
|  |  | SMA vs. AD | 0.56 | 0.11 to 1.00 | **0.015** |
| p-tau217 (ln) | **<0.001** | SMA vs. Controls | -0.09 | -0.22 to 0.05 | 0.192 |
|  |  | SMA vs. ALS | 0.32 | 0.16 to 0.49 | **<0.001** |
|  |  | SMA vs. AD | 0.45 | 0.26 to 0.63 | **<0.001** |

Table S 1: Group differences in p-tau181 and p-tau217 levels between SMA (p-tau181 n=62, p-tau217 n=64), disease controls (n=31), ALS (n=31), and AD (n=31) were assessed using multiple linear regression models. Diagnostic group was included as a categorical predictor, with age and sex as covariates. SMA was used as the reference group. β coefficients represent adjusted mean differences relative to SMA Estimates represent β coefficients for ln-transformed outcomes. Model p-values indicate the overall significance of the regression models. Pairwise p-values refer to predefined age- and sex-adjusted comparisons with SMA. AD: Alzheimer’s disease; ALS: amyotrophic lateral sclerosis; CI: confidence interval; SMA: 5q spinal muscular atrophy.

**Supplementary Table S2:** **Baseline p-tau levels across the study centres of the SMA cohort**

| Centre | n | p-tau181 [pg/mL], median (IQR) | p-tau217 [pg/mL], median (IQR) |
| --- | --- | --- | --- |
| Halle | 2 | 23.74 (12.09-35.40) | 0.24 (0.09-0.38) |
| Ulm | 29 | 16.20 (11.00-35.40) | 0.19 (0.10-0.33) |
| Leuven | 31/33 | 7.80 (4.38-20.07) | 0.11 (0.08-0.17) |

Table S 2: Distribution of p-tau181 and p-tau217 levels at baseline across the three participating centers (Halle, Ulm, and Leuven). Data are shown as median (IQR). Halle contributed only two baseline samples; therefore, no formal statistical comparison between centers was performed. IQR: interquartile range

**Supplementary Table S3:** **Comparison of baseline p-tau181 and p-tau217 levels across diagnostic groups in comparison to the Ulm cohort**

| Biomarker | Model p | Group | β (Estimate) | 95% CI | p-value |
| --- | --- | --- | --- | --- | --- |
| p-tau181 (ln) | **<0.001** | Ulm SMA vs. Controls | -0.52 | -0.73 to -0.31 | **<0.001** |
|  |  | Ulm SMA vs. ALS | 0.13 | -0.14 to 0.39 | 0.337 |
|  |  | Ulm SMA vs. AD | 0.21 | -0.08 to 0.49 | 0.156 |
| p-tau217 (ln) | **<0.001** | Ulm SMA vs. Controls | -0.14 | -0.32 to 0.04 | 0.133 |
|  |  | Ulm SMA vs. ALS | 0.25 | 0.02 to 0.48 | **0.031** |
|  |  | Ulm SMA vs. AD | 0.37 | 0.11 to 0.62 | **0.005** |

Table 3: Group differences in p-tau181 and p-tau217 levels between the Ulm SMA cohort (n=29), disease controls (n=31), ALS (n=31), and AD (n=31) were assessed using multiple linear regression models. Diagnostic group was included as a categorical predictor, with age and sex as covariates. Ulm SMA was used as the reference group. β coefficients represent adjusted mean differences relative to SMA. Estimates represent β coefficients for ln-transformed outcomes. Model p-values indicate the overall significance of the regression models. Pairwise p-values refer to predefined age- and sex-adjusted comparisons with Ulm SMA. AD: Alzheimer’s disease; ALS: amyotrophic lateral sclerosis; CI: confidence interval; SMA: 5q spinal muscular atrophy.

**Supplementary Table S4: Comparison of baseline p-tau181 and p-tau217 levels across diagnostic groups in comparison to the Leuven cohort**

| Biomarker | Model p | Group | β | 95% CI | p-value |
| --- | --- | --- | --- | --- | --- |
| p-tau181 (ln) | **<0.001** | Leuven SMA vs. Controls | -0.197 | -0.56 to 0.17 | 0.284 |
|  |  | Leuven SMA vs. ALS | 1.22 | 0.77 to 1.67 | **<0.001** |
|  |  | Leuven SMA vs. AD | 0.73 | 0.24 to 1.219 | **0.004** |
| p-tau217 (ln) | **<0.001** | Leuven SMA vs. Controls | -0.06 | -0.22 to 0.11 | 0.476 |
|  |  | Leuven SMA vs. ALS | 0.35 | 0.14 to 0.56 | **0.001** |
|  |  | Leuven SMA vs. AD | 0.47 | 0.24 to 0.699 | **<0.001** |

Table S4: Group differences in p-tau181 and p-tau217 levels between the Leuven SMA cohort (p-tau181 n=31, p-tau217 n=33), disease controls (n=31), ALS (n=31), and AD (n=31) were assessed using multiple linear regression models. Diagnostic group was included as a categorical predictor, with age and sex as covariates. Leuven SMA was used as the reference group. β coefficients represent adjusted mean differences relative to SMA. Estimates represent β coefficients for ln-transformed outcomes. Model p-values indicate the overall significance of the regression models. Pairwise p-values refer to predefined age- and sex-adjusted comparisons with Leuven SMA. AD: Alzheimer’s disease; ALS: amyotrophic lateral sclerosis; CI: confidence interval; SMA: 5q spinal muscular atrophy.

**Supplementary Table S5: Serum concentration of p-tau181 and p-tau217 at different time points in the SMA cohort**

|  | n (BL/FUP1/FUP2) | Baseline, median (IQR) | Follow-up 1, median (IQR) | Follow-up 2, median (IQR) | p-value |
| --- | --- | --- | --- | --- | --- |
| p-tau181 [pg/mL] | 62/ 37/ 59 | 11.86 (7.30-26.19) | 12.49 (5.94-19.96) | 11.57 (5.85-22.05) | 0.435 |
| p-tau217 [pg/mL] | 64/ 40/ 63 | 0.14 (0.08-0.28) | 0.12 (0.09-0.18) | 0.13 (0.09-0.24) | 0.656 |

Table S5: Data are shown as median (interquartile range). Data were analysed using the Kruskal–Wallis test based on available data per time point. BL: Baseline; FUP1: follow-up1; FUP2: follow-up2.

**Supplementary Table S6: Within-subject changes in p-tau181 and p-tau217 across study visits**

| Outcome | Comparison | n | Δ, median (IQR) | **p-value** |
| --- | --- | --- | --- | --- |
| p-tau181 [pg/mL] | Baseline vs. FUP1 | 29 | 0.17 (-7.90-5.10) | 0.983 |
|  | Baseline vs. FUP2 | 56 | -1.80 (-5.90-2.10) | **0.017** |
|  | FUP1 vs. FUP2 | 28 | 0.00 (-5.90-5.70) | 0.797 |
| p-tau217 [pg/mL] | Baseline vs. FUP1 | 35 | -0.01 (-0.04-0.03) | 0.777 |
|  | Baseline vs. FUP2 | 62 | 0.00 (-0.04-0.04) | 0.746 |
|  | FUP1 vs. FUP2 | 34 | 0.00 (-0.04-0.04) | 0.821 |

Table S6: Data are presented as median (IQR) of within-subject differences. Comparisons between study visits were performed using the Wilcoxon signed-rank test. Δ represents the difference between time points (later minus earlier visit). Sample sizes (n) indicate the number of paired observations included in each comparison. IQR: interquartile range.

**Supplementary Table S7:** **Additional clinical characteristics of the SMA cohort (n = 70), including absolute values for HFMSE and RULM, as well as MFM32 subdomain scores (%)**

|  | Overall SMA | Halle | Ulm | Leuven |
| --- | --- | --- | --- | --- |
| N | 70 | 7 | 29 | 34 |
| Baseline | 65 | 2 | 29 | 34 |
| Follow-up 1 | 40 | 6 | 0 | 34 |
| Follow-up 2 | 63 | 2 | 28 | 33 |
| HFMSE absolute, (mean ± SD)^1^ | | | | |
| Baseline | 21.95 (±21.02) | 38.5 (±23.33) | 17.38 (±20.64) | 28.07 (±20.2) |
| Follow-up 1 | 30.10 (±22.38) | 33.4 (±29.63) | - | 29.00 (±20.59) |
| Follow-up 2 | 22.85 (±21.74) | 57.00 (±0.00) | 18.36 (±21.20) | 28.07 (±20.78) |
| RULM absolute, (mean ± SD) | | | | |
| Baseline | 20.07 (±11.79) | 37.00 (±0.00) | 17.88 (±11.49) | 20.70 (±11.62) |
| Follow-up 1 | 22.90 (±11.72) | 28.49 (±11.33) | - | 22.09 (±11.72) |
| Follow-up 2 | 21.53 (±11.37) | 37.00 (±0.00) | 19.83 (±11.18) | 22.30 (±11.41) |
| MFM32% D1, (mean ± SD) ^2^ | | | | |
| Baseline | - | - | - | 7.84 (±18.95) |
| Follow-up 1 | - | - | - | 8.55 (±20.42) |
| Follow-up 2 | - | - | - | 7.54 (±17.21) |
| MFM32% D2, (mean ± SD) ^2^ | | | | |
| Baseline | - | - | - | 35.65 (±29.77) |
| Follow-up 1 | - | - | - | 38.43 (±30.25) |
| Follow-up 2 | - | - | - | 40.36 (±30.49) |
| MFM32% D3, (mean ± SD) ^2^ | | | | |
| Baseline | - | - | - | 55.26 (±32.17) |
| Follow-up 1 | - | - | - | 59.26 (±30.70) |
| Follow-up 2 | - | - | - | 64.15 (±28.87) |

Table S7: Clinical data (HFMSE, RULM, MFM32%) are displayed as mean ± standard deviation (SD) or as absolute number and percentage (n (%)) depending on the type of data1 HFMSE was collected only in nusinersen-treated patients within the Leuven cohort. 2 MFM32 was available exclusively in risdiplam-treated patients and was collected only at the Leuven center. HFMSE: Hammersmith functional motorscale expanded (HFMSE), MFM32: Motor Function Measure 32, RULM= Revised upper limb module, SD= Standard deviation.

**Supplementary Table S8: Changes in HFMSE and RULM from baseline to follow-up 2**

| **Outcome** | **n** | Baseline, median (IQR) | Follow-up 2, median (IQR) | p-value |
| --- | --- | --- | --- | --- |
| HFMSE | Baseline=40  FUP2=40 | 10.00 (4.00-38.75) | 11.50 (5.00-39.25) | 0.794 |
| RULM | Baseline=58  FUP2=57 | 19.00 (9.75-30.25) | 21.00 (13.00-33.00) | 0.367 |

Table S8: Data are presented as median (IQR). Differences between baseline and follow-up 2 were analyzed using the Mann–Whitney U test. Sample sizes reflect the number of observations available at each time point. FUP2: Follow-up 2; HFMSE: Hammersmith functional motorscale expanded (HFMSE); IQR: Interquartile Range; RULM: Revised upper limb module.

**Supplementary Table S9: Additional data for absolute values of HFMSE, RULM, and MFM32 subdomains in linear mixed-effects models**

| Predictor | Outcome | β (Estimate) | SE | 95% CI | p-value | p-value (FDR-adj.) | n |
| --- | --- | --- | --- | --- | --- | --- | --- |
| HFMSE | p-tau181 (ln) | 0.02 | 0.004 | 0.01 to 0.03 | **<0.001** | **0.001** | 101 |
|  | p-tau217 (ln) | 0.02 | 0.003 | 0.01 to 0.02 | **<0.001** | **<0.001** | 102 |
| RULM | p-tau181 (ln) | 0.05 | 0.01 | 0.03 to 0.06 | **<0.001** | **<0.001** | 147 |
|  | p-tau217 (ln) | 0.03 | 0.01 | 0.02 to 0.04 | **<0.001** | **<0.001** | 156 |
| MFM32%  D1 | p-tau181 (ln) | 0.02 | 0.01 | 0.001 to 0.04 | 0.059 | 0.110 | 45 |
|  | p-tau217 (ln) | 0.02 | 0.004 | 0.01 to 0.02 | **0.002** | **0.004** | 52 |
| MFM32%  D2 | p-tau181 (ln) | 0.02 | 0.01 | 0.02 to 0.03 | **0.001** | **0.002** | 46 |
|  | p-tau217 (ln) | 0.01 | 0.002 | 0.01 to 0.02 | **<0.001** | **<0.001** | 53 |
| MFM32%  D3 | p-tau181 (ln) | 0.02 | 0.01 | 0.01 to 0.03 | **0.004** | **0.010** | 46 |
|  | p-tau217 (ln) | 0.01 | 0.003 | 0.004 to 0.01 | **0.002** | **0.006** | 53 |

Table S9: Linear mixed-effects models with random intercept for PatientID, adjusted for timepoint, center, age, and sex. Estimates represent β coefficients for ln-transformed outcomes. P-values were adjusted using false discovery rate (FDR). MFM32 data were available only for patients treated with risdiplam and were collected exclusively at the Leuven center. CI: confidence interval; HFMSE: Hammersmith Functional Motor Scale–Expanded; MFM32: Motor Function Measure 32; D1: Domain 1: standing and transfer; D2: Domain 2: axial and proximal motor function; D3: Domain 3: distal motor function; RULM: Revised Upper Limb Module; SE: standard error.

**Supplementary Table S10:** **Creatine kinase and neurofilament light chain in the SMA cohort at different time points**

| Biomarker | Time points | Median (IQR) | n | p-value |
| --- | --- | --- | --- | --- |
| CK [U/L] | Baseline | 80.50 (32.80-208.00) | 34 | 0.926 |
|  | Follow-up 1 | 86.60 (45.30-227.00) | 38 |  |
|  | Follow-up 2 | 91.00 (34.00-184.00) | 35 |  |
| NfL [pg/mL] | Baseline | 14.80 (10.30-21.30) | 65 | **0.030** |
|  | Follow-up 1 | 10.80 (7.77-18.95) | 40 |  |
|  | Follow-up 2 | 11.10 (7.85-16.90) | 63 |  |

Table S10: Data are presented as median (IQR). Data were analyzed using the Kruskal–Wallis test, with multiple comparisons adjusted using Dunn’s multiple comparisons test. CK: creatine kinase; IQR: interquartile range; NfL: neurofilament light chain.

**Supplementary Table S11:** **Multivariable linear regression analyses of baseline p-tau181 and p-tau217 as predictors of changes in motor scores (ΔRULM and ΔHFMSE)**

|  | ΔRULM (p-tau181), β (95% CI), p | ΔRULM (p-tau217), β (95% CI), p | ΔHFMSE (p-tau181), β (95% CI), p | ΔHFMSE (p-tau217), β (95% CI), p |
| --- | --- | --- | --- | --- |
| Biomarker (ln) | 0.48 (-0.35 to 1.30), 0.253 | 0.48 (-0.66 to 1.62), 0.406 | **1.79 (0.59 to 2.99), 0.005** | 1.60 (-0.13 to 3.33), 0.068 |
| Baseline motorscore | **-0.08 (-0.15 to -0.02), 0.013** | **-0.08 (-0.15 to -0.01), 0.026** | -0.02 (-0.07 to 0.03), 0.425 | -0.08 (-0.08 to 0.04), 0.525 |
| Age | **-0.06 (-0.10 to -0.01), 0.021** | **-0.06 (-0.11 to -0.01), 0.018** | 0.21 (-0.04 to 0.08), 0.461 | 0.02 (-0.04 to 0.09), 0.497 |
| Sex (male) | **1.82 (0.48 to 3.16), 0.009** | **1.83 (0.46 to 3.20), 0.009** | 0.59 (-1.39 to 2.56), 0.548 | 0.35 (-1.77 to 2.48), 0.743 |
| R² | 0.28 | 0.27 | 0.25 | 0.13 |
| n | 52 | 54 | 37 | 37 |
| Model p | **0.004** | **0.003** | 0.053 | 0.349 |

Table S11: Values represent regression coefficients (β) with 95% confidence intervals (CI) and corresponding p-values derived from multivariable linear regression models. The dependent variable was the change in motor function between baseline and follow-up 2 (ΔRULM or ΔHFMSE). Independent variables included baseline biomarker levels (ln-transformed p-tau181 or p-tau217), baseline motor score, age, and sex (male =1, female =2). R² indicates the proportion of variance explained by the model. N represents the number of cases included in each analysis. CI: Confidence interval; HFMSE: Hammersmith Functional Motor Scale–Expanded; RULM: Revised Upper Limb Module.

**Supplementary Table S12: Linear mixed-effects models of categorical clinical variables and serum p-tau levels in SMA**

| Predictor | Outcome | Comparison (reference) | β (Estimate) | 95% CI | p-value (FDR-adj.) | n |
| --- | --- | --- | --- | --- | --- | --- |
| SMA type | p-tau181 (ln) | 1 vs. 2 | -0.59 | -1.48 to  0.31 | 0.299 | 158 |
|  | p-tau217 (ln) | 1 vs. 2 | -0.25 | -0.87 to  0.38 | 0.530 | 167 |
|  | p-tau181 (ln) | 1 vs. 3 | 0.41 | -0.50 to  1.32 | 0.484 | 158 |
|  | p-tau217 (ln) | 1 vs. 3 | 0.38 | -0.26 to  1.02 | 0.333 | 167 |
|  | p-tau181 (ln) | 1 vs. 4 | 1.15 | 0.00 to  2.30 | 0.106 | 158 |
|  | p-tau217 (ln) | 1 vs. 4 | 0.90 | 0.09 to  1.71 | 0.066 | 167 |
| Functional status | p-tau181 (ln) | Ambulatory with walking aids vs. non-ambulatory | 1.56 | 0.76 to  2.37 | **0.001** | 102 |
|  | p-tau217 (ln) | Ambulatory with walking aids vs. non-ambulatory | 0.66 | 0.21  to 1.11 | **0.011** | 110 |
|  | p-tau181 (ln) | Ambulatory vs. non-ambulatory | 1.33 | 0.82 to  1.85 | **<0.001** | 102 |
|  | p-tau217 (ln) | Ambulatory vs. non-ambulatory | 0.93 | 0.65 to  1.21 | **<0.001** | 110 |

Table S12: Linear mixed-effects models with random intercept for PatientID, adjusted for timepoint, center, age, and sex were using analysing the association between between categorical clinical variables and ln-transformed p-tau181 and p-tau217 levels. β coefficients represent adjusted mean differences relative to the reference group. For SMA type, type I was used as the reference category; for functional status, non-ambulatory patients served as the reference group. P-values were adjusted using false discovery rate (FDR). CI: confidence interval.

**Supplementary Table S13: Demographic and clinical data of follow-up 3 (n=8)**

| n | 8 |
| --- | --- |
| Age [years], median (IQR) | 50.00 (38.50-63.50) |
| Female, n (%) | 2 (25) |
| SMA type, n (%) |  |
| I | 0 |
| II | 1 (12.5) |
| III | 6 (75) |
| IV | 1 (12.5) |
| SMN2 copy number, n (%) |  |
| 2 | 0 |
| 3 | 1 (12.5) |
| 4 | 3 (37.5) |
| 4+ | 1 (12.5) |
| unknown | 3 (37.5) |
| Therapy, n (%) |  |
| Risdiplam | 0 |
| Nusinersen | 8 (100) |
| Disease duration [years], (mean ± SD) | 44 (±15.56) |
| HFMSE absolute, (mean ± SD) | 40.29 (±18.42) |
| HFMSE%, (mean ± SD) | 61.04 (±27.91) |
| RULM absolute, (mean ± SD) | 31 (±8.54) |
| RULM%, (mean ± SD) | 83.78 (±23.09) |
| FVC [% predicted], (mean ± SD) | 99 (±24.62) |

Table S13: Eight patients from a single center (Halle) were included; results should therefore be considered preliminary. Age is presented as median and interquartile range (IQR), sex, and clinical characteristics (SMA type, SMN2 copy number, treatment, disease duration, HFMSE, IQR: interquartile range, RULM, and FVC % predicted) are presented as mean ± standard deviation (SD) or as absolute numbers and percentages (n, %), as appropriate. FVC: forced vital capacity; HFMSE: Hammersmith Functional Motor Scale–Expanded; RULM: Revised Upper Limb Module; SD: standard deviation.

**Supplementary Table S14: p-tau181 and p-tau217 at follow-up 3 (n=8)**

| Biomarker | Median (IQR) |
| --- | --- |
| p-tau181 [pg/mL] | 42.67 (19.95-49.15) |
| p- tau217 [pg/mL] | 0.35 (0.09-0.47) |

Table S14: Data are presented as median (IQR). Due to the small sample size no statistical testing was performed.

**Supplementary Table S15: Spearman correlations between body mass index (BMI) and serum p-tau levels**

| Outcome | n | Spearman r | p-value |
| --- | --- | --- | --- |
| BMI vs. p-tau181 | 33 | 0.12 | 0.544 |
| BMI vs. p-tau217 | 35 | 0.14 | 0.413 |

Table S15: Spearman correlation coefficients (ρ) are shown for patients with available BMI data. BMI: body mass index.

**Supplementary Table S16: Serum concentration of NfL at different time points in the SMA cohort**

| Comparison | Mean Rank Difference | Adj. p-value |
| --- | --- | --- |
| Baseline vs. Follow-up 1 | 19.50 | 0.138 |
| Baseline vs. Follow-up 2 | 20.91 | **0.045** |
| Follow-up 1 vs. Follow-up 2 | 1.42 | >0.999 |

Table S16: Data were analyzed using the Kruskal–Wallis test based on available data per time point with multiple comparisons adjusted using Dunn’s multiple comparisons test. Baseline: n=65, Follow-up 1: n= 40, Follow-up 2: n=63. NfL: Neurofilament light chain.

**SUPPLEMENTARY FIGURES**

**Supplementary Fig. S1: Longitudinal distribution of serum p-tau181 and p-tau217 levels in SMA**

Figure S1: a: p-tau181 at baseline (n=62), follow-up 1 (n=37) and follow-up 2 (n=59), follow-up 3 (n=8). b: p-tau217 at baseline (n=64), follow-up 1 (n=40) and follow-up 2 (n=63), follow-up 3 (n=8). Plots depict the distribution of biomarker levels at different time points, with medians and interquartile ranges indicated; individual data points are overlaid. Due to the small sample size at follow-up 3, no statistical testing was performed for this time point. The y-axis is displayed on a logarithmic (base-10) scale.

**Supplementary Fig. S2: Forest plot of associations between CK and NfL (both ln-transformed) and plasma p-tau181 and p-tau217 levels in SMA**

Figure S2: Forest plot showing β coefficients with 95% confidence intervals derived from linear mixed-effects models examining associations between ln-transformed CK and NfL and ln-transformed plasma p-tau181 and p-tau217 levels. Linear mixed-effects models with random intercept for PatientID, adjusted for timepoint, center, age, and sex. Effect estimates represent the change in ln(p-tau) per one-unit increase in the respective predictor. The vertical reference line indicates no association (β = 0). P-values were adjusted for multiple testing using false discovery rate (FDR) correction. CI: Confidence interval; CK: creatine kinase; FDR: false discovery rate; NfL: neurofilament light chain.

**Supplementary Figure S3: NfL levels in the SMA cohort at different time points**

Figure S3: NfL at baseline (n=65), follow-up 1 (n=40) and follow-up 2 (n=63). Plots depict the distribution of biomarker levels in patients at different time points, with medians and interquartile ranges indicated; individual data points are overlaid. Group comparisons across time points were performed using the Kruskal–Wallis-test based on available data per time point with Dunnn’s multiple comparison adjustment. The y-axis is displayed on a logarithmic (base-10) scale. FUP1: Follow-up 1; FUP2: Follow-up 2; NfL: Neurofilament light chain.

**REFERENCES**

[1]	S. Abu-Rumeileh *et al.*, “Phosphorylated tau 181 and 217 are elevated in serum and muscle of patients with amyotrophic lateral sclerosis,” *Nature Communications* , vol. 16, no. 1, Dec. 2025, doi: 10.1038/s41467-025-57144-7.
